# Relationship between CTX-II and patient characteristics, patient-reported outcome, muscle strength, and rehabilitation in patients with a focal cartilage lesion of the knee: a prospective exploratory cohort study of 48 patients

**DOI:** 10.1186/1471-2474-15-99

**Published:** 2014-03-24

**Authors:** Jan Harald Røtterud, Finn P Reinholt, Karen Johanne Beckstrøm, May Arna Risberg, Asbjørn Årøen

**Affiliations:** 1 Department of Orthopedic Surgery, Akershus University Hospital, Lørenskog, Norway; 2 Institute of Clinical Medicine, Akershus University Hospital, University of Oslo, Lørenskog, Norway; 3 Department of Pathology, Oslo University Hospital, Oslo, Norway; 4 Institute of Pathology, University of Oslo, Oslo, Norway; 5 Institute of Immunology, Oslo University Hospital, Oslo, Norway; 6 Norwegian Center for Stem Cell Research, Institute of Basic Medical Sciences, University of Oslo, Oslo, Norway; 7 Norwegian Research Center for Active Rehabilitation (NAR), Department of Sport Medicine, Norwegian School of Sport Sciences, Oslo, Norway; 8 Department of Orthopaedics, Oslo University Hospital, Oslo, Norway; 9 Oslo Sports Trauma Research Center, Oslo, Norway

**Keywords:** Knee, Cartilage lesion, CTX-II, Rehabilitation, Muscle strength, KOOS

## Abstract

**Background:**

C-telopeptide fragments of type II collagen (CTX-II) are created during articular cartilage breakdown and CTX-II is considered useful as a biomarker of osteoarthritis. The primary objective of the present study was to explore the relationship between urinary CTX-II concentration and patient characteristics, patient-reported outcome, muscle strength, and rehabilitation in patients with isolated focal knee cartilage lesions. Furthermore, the secondary objective was to examine differences in urinary CTX-II concentration between patients with focal cartilage lesions and healthy controls.

**Methods:**

48 patients (mean age 33.4 years, standard deviation 9.0) with a focal full-thickness (International Cartilage Repair Society grade 3 or 4) cartilage lesion on the medial or lateral femoral condyle were included. After baseline assessments, the patients completed a 3-month rehabilitation program and 44 patients attended the 3 month follow-up. Baseline and follow-up assessments consisted of urinary CTX-II, the Knee Injury and Osteoarthritis Outcome Score (KOOS), and isokinetic quadriceps and hamstring muscle strength measurements. CTX-II was also analysed in urine samples from 6 healthy individuals, serving as normal controls. Correlations were classified as very weak (correlation coefficient [r] < 0.20), weak (r = 0.20 – 0.39), moderate (r = 0.40 – 0.59), strong (r = 0.60 – 0.79), and very strong (r > 0.80).

**Results:**

Except for age and quadriceps strength, no significant correlations were found between CTX-II concentrations and baseline characteristics, KOOS, or muscle strength. Except for age, all correlations were considered as weak or very weak. The patients with a focal cartilage lesion had significantly higher mean CTX-II concentration than the healthy control individuals both at baseline (p = 0.001) and at follow-up (p = 0.001). The mean CTX-II concentration tended to decrease during rehabilitation, but the reduction was not significant (p = 0.076).

**Conclusions:**

The current exploratory study demonstrated that patients with a focal cartilage lesion of the knee had higher concentrations of urinary CTX-II than healthy individuals. In addition, CTX-II concentration tended to decrease during rehabilitation.

**Trial registration:**

ClinicalTrials.gov NCT00885729

## Background

Focal cartilage lesions of the knee are a common finding in arthroscopic surgery [1] and may lead to debilitating knee complaints [2]. Even though focal cartilage lesions of the knee are considered as a possible risk factor for later osteoarthritis (OA) [3,4], the natural history of these lesions is not well understood.

The development of OA is traditionally assessed by plain radiographs. However, radiographic changes develop over relatively long periods of time and occur in late stages of OA. Magnetic resonance imaging (MRI), including techniques such as d’GEMRIC, T2-mapping, and T1-rho, can also be used to assess both quantitative and qualitative changes of cartilage [5]. However, these are expensive and time-consuming methods. A third option in monitoring OA development is joint tissue-related biomarkers produced during synthesis or degradation of articular cartilage.

One such biomarker is C-telopeptide fragments of type II collagen (CTX-II) created during articular cartilage breakdown and excreted in urine [6]. Urinary CTX-II has been shown to be elevated in correlation with the presence and severity of knee OA [7-9] and to correlate with joint loading and exercise in patients with knee OA [10]. In addition, decreasing CTX-II concentrations after anterior cruciate ligament reconstruction have been associated with decreasing knee pain and improving function [11]. Consequently, there might be a potential of using CTX-II in assessing the development of degenerative changes and treatment effects also in patients with focal cartilage lesions of the knee. However, most studies on CTX-II have focused on patients with knee OA and not on patients with focal cartilage lesions. Knowledge from studies on knee OA patients can not be directly transferred to patients with focal cartilage lesions, as they differ from each other, especially regarding patient age.

If CTX-II is to be used in monitoring treatment effects and natural history of cartilage lesions, there is a need for knowledge on the relationship between CTX-II concentrations and various patient-related clinical features and changes in CTX-II concentrations following rehabilitation in patients with cartilage lesions. Thus, the purpose of the present study was to explore these issues in a cohort of patients with focal knee cartilage lesions undergoing a 3-month rehabilitation program. The primary objective was to investigate the relationship between CTX-II concentrations and age, gender, Body Mass Index (BMI), duration of symptoms, previous ipsilateral knee surgery, size of the cartilage lesion, knee muscle strength, and patient-reported outcome measures in patients with focal knee cartilage lesions. The secondary objectives were to examine if the patients showed different CTX-II concentrations than healthy control individuals and to examine changes in the patients’ CTX-II concentrations following a 3-month rehabilitation program.

## Methods

### Patients and procedures

The present study was a part of the Oslo Cartilage Active Rehabilitation and Education Study (The Oslo CARE study) on rehabilitation of patients with focal cartilage lesions of the knee [12]. The study was a prospective cohort study, and the inclusion criteria were age between 18 to 50 years, an arthroscopically verified focal cartilage lesion of International Cartilage Repair Society (ICRS) grade 3 or 4 on the medial or lateral femoral condyle of at least 1.5 cm in diameter and less than 6.0 cm^2^, and Lysholm score [13] less than 75. Lesion size was estimated using a standard 4-mm arthroscopic probe, measuring the longest anteroposterior distance and the width of the cartilage lesion. Exclusion criteria were OA, untreated meniscus lesion, major ligament injury, and major malalignment. The presence of OA was evaluated radiographically [14] according to the Kellgren-Lawrence (K-L) classification by the including orthopedic surgeon, [15] and OA was defined as K-L grade ≥ 2.

Prior to inclusion, all patients signed a written informed-consent form. The study was conducted in accordance with the Declaration of Helsinki, approved by The South-Eastern Norway Regional Ethical Committee and registered at ClinicalTrials.gov (NCT00885729).

Patients were recruited consecutively from a cartilage centre at a university hospital and 48 out of 50 eligible patients were included in the study. All patients went through a diagnostic arthroscopy prior to inclusion.

Baseline testing was performed at a minimum of 6 weeks after the diagnostic arthroscopy. All patients (n = 48) completed a test session which included collection of urine samples, patient-reported outcome measures, and isokinetic muscle strength testing. After the baseline testing, the patients attended a 3-month active rehabilitation program consisting of cardiovascular training, knee and hip progressive resistance training, and neuromuscular training. The rehabilitation program and its feasibility have been described by Wondrasch et al. [12], and showed a high adherence rate, clinically meaningful changes of patient-reported outcomes, positive load progression, and the occurrence of very few joint-specific adverse events [12]. After completing the rehabilitation program at a median of 3.1 months (range, 2.8-8.7), 44 patients attended a follow-up test session and conducted the same assessments as the baseline testing. 4 patients did not attend the 3-month follow-up and were considered lost to follow-up.

### Variables

#### Baseline characteristics

Of the 48 patients included, 34 were male. Mean age was 33.4 years (standard deviation [SD], 9.0) and mean Body Mass Index (BMI) 27.3 (SD, 4.9). Median duration of symptoms was 27 months (range, 2-240), 24 patients related their debut of symptoms to a knee trauma and 13 patients had a history of osteochondritis dissecans. Median number of previous ipsilateral knee surgeries was 2 (range, 0-4). In 37 cases the cartilage lesion was located on the medial femoral condyle, and in the 11 remaining cases the lesion was located on the lateral femoral condyle. The mean size of the cartilage lesion was 2.9 cm^2^ (SD, 1.3). There were no differences in baseline characteristics between the patients lost to follow-up (n = 4) and the rest of the patients (n = 44) (data not shown).

#### CTX-II

Urine samples were collected during daytime just prior to the physical testing, and stored at -70°C until analysis. CTX-II concentrations were determined using an enzyme immunoassay (EIA) kit based on a mouse monoclonal antibody against the EKGPDP sequence of human type II collagen C-telopeptide (Urine CartiLaps® EIA; Nordic Bioscience, Herlev, Denmark). Urine samples were diluted as needed, ranging from 1:1 to 1:50. CTX-II concentrations were corrected for urinary creatinine concentration as recommended by the producer, using the formula (corrected CTX-II concentration [ng/mmol] = 1000 × urine CartiLaps [µg/L]/creatinine [mmol/L]).

#### Knee Injury and Osteoarthritis Outcome Score (KOOS)

KOOS is a self-administered knee-specific questionnaire, validated for patients with several types of knee injury, knee OA, and for patients with cartilage lesions [16-18]. KOOS consists of 42 questions distributed in 5 subscales: Pain (9 questions), Symptoms (7 questions), Activities in Daily Living (ADL) (17 questions), Function in Sport and Recreation (Sport/Rec) (5 questions) and Knee-Related Quality of Life (QoL) (4 questions). Each subscale is scored individually on a scale ranging from 0 (worst) to 100 (best) [19].

The results from the KOOS reported by the current patient cohort at baseline and at follow-up after 3 month rehabilitation have previously been published by Wondrasch et al. [12], and are referred to here as descriptives. The mean KOOS values at baseline (n = 48) were: Pain 54.8 (SD, 17.7), Symptoms 59.0 (SD, 17.0), ADL 70.1 (SD, 17.6), Sport/rec 27.5 (SD, 20.6), and QoL 27.6 (SD, 16.5). At follow-up (n = 44) after 3-month rehabilitation, the mean KOOS values were: Pain 60.1 (SD, 17.8), Symptoms 64.9 (SD, 15.9), ADL 75.6 (SD, 17.0), Sport/rec 41.0 (SD, 25.4), and QoL 39.4 (SD, 20.6).

#### Isokinetic muscle strength

Isokinetic muscle strength testing for the quadriceps and the hamstrings were performed with an isokinetic dynamometer (Biodex 6000; Biodex Medical Systems, Inc, Shirley, NY). Prior to testing, the patients performed a 10-minute warm-up on a stationary bike. The test was conducted using 5 repetitions at an angular velocity of 60°/s and muscle strength was quantified based on peak torque (Nm).

The results from the isokinetic quadriceps and hamstrings muscle strength measurements of the current study cohort at baseline and at follow-up after 3 month rehabilitation have previously been published by Wondrasch et al. [12], and are referred to here as descriptives. The mean quadriceps strength of the injured leg at baseline (n = 48) was 139.7 Nm (SD 63.9), and at follow-up (n = 44) after 3 months 178.2 Nm (SD 59.6). At baseline, this represented a mean of 74.2% (SD, 27.2) of the quadriceps strength of the uninjured limb, and at follow-up 88.4% (SD, 18.1). The mean hamstrings strength of the injured leg at baseline (n = 48) was 77.9 Nm (SD, 38.6), and at follow-up (n = 44) 97.4 Nm (SD, 28.9). At baseline, this represented a mean of 84.1% (SD, 36.5) of the hamstrings strength of the uninjured limb, and at follow-up 93.0% (SD, 15.8).

### Controls

For CTX-II analysis, urine samples were collected from 6 healthy individuals with no record of knee problems or major knee injury. These were 5 males and 1 female, mean age 35.7 years (SD, 2.3). The urine samples from these individuals were collected at one time point only, as they did not attend the rehabilitation program, and served as normal controls for both the baseline and follow-up patient CTX-II concentrations.

### Statistical analysis

All analyses were performed using Statistical Package of Social Sciences (SPSS) software version 20 (SPSS, Inc, an IBM Company, Chicago, Illinois, US). All CTX-II concentrations were log transformed to reduce the effect of outliers. For correlations between CTX-II and baseline variables and outcome measures, Pearson correlation coefficients were calculated. Correlations were classified as very weak (correlation coefficient [r] < 0.20), weak (r = 0.20 – 0.39), moderate (r = 0.40 – 0.59), strong (r = 0.60 – 0.79), and very strong (r > 0.80). The Mann-Whitney U test was used to compare the mean CTX-II concentrations between patients and controls. Paired samples t-test was used to compare the mean CTX-II concentrations pre and post rehabilitation. The level of significance was defined as p ≤ 0.05.

## Results

### CTX-II

At baseline (n = 48) the mean CTX-II concentration of the patients with a focal cartilage lesion of the knee was 579 ng/mmol (95% CI, 266 to 891) (log 2.52 [95% CI, 2.41 to 2.63]). At follow-up (n = 44) after 3-month rehabilitation period there was a decrease to 514 ng/mmol (95% CI, 278 to 750) (log 2.50 [95% CI, 2.38 to 2.61]). The difference in mean CTX-II concentration from baseline to follow-up (n = 44), -102 (95% CI, -236 to 33) (log –0.05 [95% CI, -0.11 to 0.01]), was not statistically significant (p = 0.076).

The healthy control individuals (n = 6) had a mean CTX-II concentration of 129 ng/mmol (95% CI, 111.2 to 146.8) (log 2.11 [95% CI, 2.04 to 2.17]). The patients with a focal cartilage lesion showed a significantly higher mean concentration of CTX-II than the healthy control individuals both at baseline (p = 0.001) and at follow-up (p = 0.001).

### CTX-II and baseline characteristics

Correlations analysis between the baseline characteristics (age, gender, BMI, duration of symptoms, previous knee surgery, and size of cartilage lesion) and CTX-II at baseline, follow-up, and change from baseline to follow-up revealed no significant correlations except for age (Table 1). Except for age, all correlations were considered as weak or very weak.

**Table 1:** **Correlations between baseline characteristics and CTX-II**^**a **^**at baseline, at follow-up and change from baseline to follow-up**

	**CTX-II at baseline**			**CTX-II at follow-up**			**Δ**^**b**^**CTX-II**		
**Baseline characteristic**	**n**	**r**^**c**^	**p**^**d**^	**n**	**r**^**c**^	**p**^**d**^	**n**	**r**^**c**^	**p**^**d**^
Age	48	-0.566	0.001	44	-0.694	<0.001	44	-0.199	ns
Gender	48	0.205	ns	44	0.187	ns	44	-0.069	ns
Body Mass Index	48	-0.247	ns	44	-0.151	ns	44	0.201	ns
Duration of symptoms	48	-0.088	ns	44	-0.086	ns	44	0.016	ns
Previous knee surgery	48	0.060	ns	44	-0.018	ns	44	0.005	ns
Size of cartilage lesion	46	0.100	ns	43	0.065	ns	43	-0.137	ns

^a^Log transformed values; ^b^Δ, change from baseline to follow-up; ^c^r, Pearson correlation coefficient; ^d^p, level of significance; ns, not significant.

### CTX-II and KOOS

Correlation analysis between KOOS at baseline and CTX-II at baseline, between KOOS at follow-up and CTX-II at follow-up, and between change in KOOS from baseline to follow-up and change in CTX-II from baseline to follow-up revealed no significant correlations (Table 2). All correlations were considered as weak or very weak.

**Table 2:** **Correlations between CTX-II**^**a **^**and Knee Injury and Osteoarthritis Outcome Score (KOOS) at baseline, follow-up and change from baseline to follow-up**

	**Baseline CTX-II vs. baseline KOOS (n = 48)**		**Follow-up CTX-II vs. follow-up KOOS (n = 44)**		**Δ**^**b**^**CTX-II vs. Δ**^**b**^**KOOS (n = 44)**	
	**r**^**c**^	**p**^**d**^	**r**^**c**^	**p**^**d**^	**r**^**c**^	**p**^**d**^
KOOS Pain	-0.093	ns	0.091	ns	0.078	ns
KOOS Symptoms	-0.140	ns	0.082	ns	0.251	ns
KOOS ADL^e^	-0.190	ns	0.124	ns	0.058	ns
KOOS Sport/Rec	-0.161	ns	0.199	ns	-0.077	ns
KOOS QoL^f^	-0.088	ns	0.060	ns	-0.114	ns

^a^Log transformed values; ^b^Δ, change from baseline to follow-up; ^c^r, Pearson correlation coefficient; ^d^p, level of significance; ns, not significant; ^e^ADL, Activity of Daily Living; ^f^QoL, Quality of Life.

### CTX-II and muscle strength

Correlation analysis between isokinetic muscle strength at baseline and CTX-II at baseline, between isokinetic muscle strength at follow-up and CTX-II at follow-up, and between change in isokinetic muscle strength from baseline to follow-up and change in CTX-II from baseline to follow-up revealed no significant correlations except for quadriceps strength at baseline (Table 3). All correlations were considered as weak or very weak.

**Table 3:** **Correlations between CTX-II**^**a **^**and isokinetic muscle strength (IMS) at baseline, follow-up and change from baseline to follow-up**

	**Baseline CTX-II vs. baseline IMS (n = 48)**		**Follow-up CTX-II vs. follow-up IMS (n = 44)**		**Δ**^**b**^**CTX-II vs. Δ**^**b**^**IMS (n = 44)**	
	**r**^**c**^	**p**^**d**^	**r**^**c**^	**p**^**d**^	**r**^**c**^	**p**^**d**^
Quadriceps, injured limb	-0.297	0.040	-0.081	ns	-0.054	ns
Quadriceps, %^e^	-0.299	0.039	0.069	ns	-0.012	ns
Hamstrings, injured limb	-0.176	ns	-0.045	ns	0.150	ns
Hamstrings, %^e^	-0.148	ns	-0.003	ns	0.069	ns

^a^Log transformed values; ^b^Δ, change from baseline to follow-up; ^c^r, Pearson correlation coefficient; ^d^p, level of significance; ^e^%, isokinetic muscle strength injured limb in percentage of isokinetic muscle strength uninjured limb; ns, not significant.

## Discussion

The present study demonstrated that patients with focal cartilage lesions of the knee had higher CTX-II concentrations compared with healthy individuals. In addition, there was a trend toward a reduction in CTX-II concentration among patients with focal knee cartilage lesions after completing a 3 month rehabilitation program. Furthermore, the present study revealed, except for age, no clinically relevant relationships between CTX-II concentrations and a variety of clinical features in patients with focal knee cartilage lesions. These findings add new information, as the present study, to our knowledge is the first to investigate urinary CTX-II concentrations among patients with isolated focal knee cartilage lesions. Previous studies on CTX-II and knee problems have primarily focused on patients with knee OA [7-10,20-22], in addition to one study on patients with anterior cruciate ligament reconstruction [11].

It has previously been shown in a study of 650 healthy individuals with age between 20 to 87 years that CTX-II concentrations vary considerably with age [23]. In line with this study, the present study revealed a significant and moderate to strong correlation between age and CTX-II concentrations, also in this relatively young patient population with focal cartilage lesions.

A previous study by Ding et al. [20] showed that knee cartilage lesion severity was significantly associated with CTX-II concentration, which is in contrast to the present study, where no correlation between cartilage lesion size and CTX-II concentration was found. However, these findings are difficult to compare, as the cohort of patients in the two studies were different. The patients in the study by Ding et al. were older (mean age 45 years), they had radiographic OA, and the cartilage lesions were classified by MRI, while the patients in the present study were younger (mean age 33 years), they had no radiographic OA, and the cartilage lesions were classified arthroscopically.

Even though a statistically significant correlation between CTX-II and quadriceps strength was found in the present study, the correlation was classified as weak and therefore not considered clinically relevant. Hence, except for age, no clinically relevant relationships between CTX-II and the clinical features studied (gender, BMI, duration of symptoms, previous knee surgery, patient-reported outcome, and muscle strength) were found in the present study. Based on these results, the value of using CTX-II on an individual basis to characterize patients with focal knee cartilage lesions will be limited.

The mean CTX-II concentration found among the patients in the present study were 579 ng/mmol, which is on a similar or even a higher level than CTX-II concentrations found in previous studies on patients with OA. Sowers et al. [7] found a mean CTX-II concentration of 345 ng/mmol in 20 patients with severe knee OA (K-L grade 3-4), and Jung et al. [24] reported a mean concentration of 429 ng/mmol in 37 patients with knee OA. Furthermore, in the present study the CTX-II concentrations among the patients with focal cartilage lesions were elevated compared to the healthy individuals. This elevation is an indication of an increased level of cartilage degradation. Even though patients with radiographic or arthroscopically diagnosed OA were excluded from the present study, the CTX-II concentrations were raised compared to the healthy individuals. Whether this raise originates from cartilage degradation directly in the cartilage lesion, from its nearby surroundings, and/or from a more generalized degradation process in the knee, like pre-radiographic OA, is not possible to differentiate in this study. Moreover, the possibility of leakage of newly synthesized collagen from the lesion area can not be ruled out. However, since the present study proposes that focal cartilage lesions affect CTX-II concentration, there might be a potential of using CTX-II in the evaluation of the natural history of such lesions.

In the present study there was a trend toward a reduction in CTX-II concentration among patients with focal knee cartilage lesions after completing a 3-month rehabilitation program. This trend could be related to a spontaneous healing of the lesion or just due to variations over time, however, it could also indicate a possible positive effect of rehabilitation on cartilage. The latter is supported by a previous study on cartilage biomarkers and effects of exercise in patients with knee OA, which suggested a potential beneficial role of exercise on cartilage structure [10].

The strengths of the present study were the prospective design and the low rate of patients lost to follow-up, combined with a high adherence rate to the rehabilitation program [12].

On the other hand, the present study has several limitations. The patients were recruited from a cartilage centre and may not be representative of a broad population of patients with focal cartilage lesions of the knee [25]. There was no randomization or actual control group. Even though CTX-II concentrations were measured in 6 healthy individuals acting as controls, this group was small and only matched for age. Among the controls, CTX-II concentrations were measured at only one time point, and they did not attend the rehabilitation program, perform any of the muscle strength testing or answer the KOOS questionnaire. Additionally, since the power analysis was done for another part of the study [12], a priori power calculation was not based on CTX-II as the outcome measure of interest. This is a major limitation, and considering the skewness in the confidence interval for the difference between mean CTX-II concentrations at baseline and follow-up, the present study might be underpowered. For further studies on CTX-II and treatment effects in patients with focal cartilage lesions we recommend that the findings from the present exploratory study are taken into account when calculating sample size.

Due to the limitations of the present exploratory study, further and improved studies on CTX-II in patients with focal cartilage lesions are needed before it can be decided whether urinary CTX-II concentration can be used in the evaluation of treatment effects and natural history of these lesions.

## Conclusions

The present exploratory study showed that patients with focal cartilage lesions of the knee had higher concentrations of CTX-II than healthy individuals. Although statistically not significant, CTX-II concentrations tended to decrease during rehabilitation of these patients. Moreover, except for age, no clinically relevant relationships between CTX-II and a variety of clinical features in patients with focal knee cartilage lesions were observed.

## Competing interests

The authors declare that they have no competing interests.

## Authors’ contributions

JHR participated in the design, collected data, performed the statistical analysis, interpreted the data, and drafted the manuscript; FPR participated in the design and data interpretation; KJB analyzed the urine samples and calculated the CTX-II concentrations; MAR participated in the design, data collection, and data interpretation; AÅ designed the study, collected data, and participated in the data interpretation. All authors reviewed the manuscript and approved the final version.

## Pre-publication history

The pre-publication history for this paper can be accessed here:

http://www.biomedcentral.com/1471-2474/15/99/prepub
